# Prediction of conformational epitopes with the use of a knowledge-based energy function and geometrically related neighboring residue characteristics

**DOI:** 10.1186/1471-2105-14-S4-S3

**Published:** 2013-03-08

**Authors:** Ying-Tsang Lo, Tun-Wen Pai, Wei-Kuo Wu, Hao-Teng Chang

**Affiliations:** 1 Department of Computer Science and Engineering, National Taiwan Ocean University, Keelung, Taiwan, R.O.C; 2 Center of Excellence for Marine Bioenvironment and Biotechnology, National Taiwan Ocean University, Keelung, Taiwan, R.O.C; 3 Graduate Institute of Molecular Systems Biomedicine, China Medical University, Taichung, Taiwan, R.O.C; 4 China Medical University Hospital, Taichung, Taiwan, R.O.C

## Abstract

**Background:**

A conformational epitope (CE) in an antigentic protein is composed of amino acid residues that are spatially near each other on the antigen's surface but are separated in sequence; CEs bind their complementary paratopes in B-cell receptors and/or antibodies. CE predication is used during vaccine design and in immuno-biological experiments. Here, we develop a novel system, CE-KEG, which predicts CEs based on knowledge-based energy and geometrical neighboring residue contents. The workflow applied grid-based mathematical morphological algorithms to efficiently detect the surface atoms of the antigens. After extracting surface residues, we ranked CE candidate residues first according to their local average energy distributions. Then, the frequencies at which geometrically related neighboring residue combinations in the potential CEs occurred were incorporated into our workflow, and the weighted combinations of the average energies and neighboring residue frequencies were used to assess the sensitivity, accuracy, and efficiency of our prediction workflow.

**Results:**

We prepared a database containing 247 antigen structures and a second database containing the 163 non-redundant antigen structures in the first database to test our workflow. Our predictive workflow performed better than did algorithms found in the literature in terms of accuracy and efficiency. For the non-redundant dataset tested, our workflow achieved an average of 47.8% sensitivity, 84.3% specificity, and 80.7% accuracy according to a 10-fold cross-validation mechanism, and the performance was evaluated under providing top three predicted CE candidates for each antigen.

**Conclusions:**

Our method combines an energy profile for surface residues with the frequency that each geometrically related amino acid residue pair occurs to identify possible CEs in antigens. This combination of these features facilitates improved identification for immuno-biological studies and synthetic vaccine design. CE-KEG is available at http://cekeg.cs.ntou.edu.tw.

## Introduction

A B-cell epitope, also known as an antigenic determinant, is the surface portion of an antigen that interacts with a B-cell receptor and/or an antibody to elicit either a cellular or humoral immune response [1,2]. Because of their diversity, B-cell epitopes have a huge potential for immunology-related applications, such as vaccine design and disease prevention, diagnosis, and treatment [3,4]. Although clinical and biological researchers usually depend on biochemical/biophysical experiments to identify epitope-binding sites in B-cell receptors and/or antibodies, such work can be expensive, time-consuming, and not always successful. Therefore, *in silico *methods that can reliably predict B-cell epitopes would simplify immunology-related experiments [5]. Given accurate epitope-prediction tools, immunologists can then focus on the appropriate protein residues and reduce their experimental efforts.

In general, epitopes are described as linear (continuous) or conformational (discontinuous) [6]. A linear epitope (LE) is a short, continuous sequence of amino acid residues on the surface of an antigen. Although an isolated LE is usually flexible, which destroys any information concerning its conformation in the protein, it can adapt that conformation to react weakly with a complementary antibody. Conversely, a conformational epitope (CE) is composed of residues that are not sequential but are near in space [7]. Several algorithms, which require a protein sequence as input, are available for LE prediction, including BEPITOPE [8], BCEPred [9], BepiPred [10], ABCpred [11], LEPS [12,13] and BCPreds [14]. These algorithms assess the physicochemical propensities, such as polarity, charge, or secondary structure, of the residues within the targeted protein sequence, and then apply quantitative matrices or machine-learning algorithms, such as the hidden Markov model, a support vector machine algorithm, or an artificial neural network algorithm, to predict LEs. However, the number of LEs on native proteins has been estimated to be ~10% of all B-cell epitopes, and most B-cell epitopes are CEs [15]. Therefore, to focus on the identification of CEs is the more practical and valuable task. For CE prediction, several algorithms have been developed including CEP [16], DiscoTope [17], PEPOP [18], ElliPro [19], PEPITO [20], and SEPPA [21], all of which use combinations of the physicochemical characteristics of known epitope residues and trained statistical features of known antigen-antibody complexes to identify CE candidates.

A different approach relies on phage display to produce peptide mimotopes that can be used to characterize the relationship between an epitope and a B-cell receptor or an antibody. Peptide mimotopes bind B-cell receptors and antibodies in a manner similar to those of their corresponding epitopes. LEs and CEs can be identified by mimotope phage display experiments. MIMOP is a hybrid computational tool that predicts epitopes from information garnered from mimotope peptide sequences [22]. Similarly, Mapitope and Pep-3D-Search use mimotope sequences to search linear sequences for matching patterns of structures on antigen surfaces. Other algorithms can identify CE residues with the use of the Ant Colony Optimization algorithm and statistical threshold parameters based on nonsequential residue pair frequencies [23,24].

Crystal and solution structures of the interfaces of antigen-antibody complexes characterize the binding specificities of the proteins in terms of hydrogen bond formation, van der Walls contacts, hydrophobicity and electrostatic interactions (reviewed by [25]). Only a small number residues located within the antigen-antibody interface energetically contribute to the binding affinity, which defines these residues as the "true" antigenic epitope [26]. Hence, we hypothesized that the energetically important residues in epitopes could be identified *in silico*. We assumed that the free, overall native antigen structure is the lowest free energy state, but that residues involving in antibody binding would possess higher potential energies. Two types of potential energy functions are currently used for energy calculations involving proteins: a physical-based potential function that focuses on the fundamental forces between atoms, and a knowledge-based potential that relies on parameters derived from experimentally solved protein structures [27]. Owing to the heavy computational complexity required for the first approach, we adopted the knowledge-based potential for our workflow. The energy functions for the surface residues used are those of the Protein Structure Analysis website [28].

Additionally, a study concerning LE prediction [29] showed that certain sequential residue pairs occur more frequently in LE epitopes than in non-epitopes. A similar statistical feature may, therefore, enhance the performance of a CE prediction workflow. Hence, we incorporated the statistical distribution of geometrically related pairs of residues found in verified CEs and the identification of residues with relatively high energy profiles. We first located surface residues with relatively high knowledge-based energies within a specified radius of a sphere and assigned them as the initial anchors of candidate epitope regions. Then we extended the surfaces to include neighboring residues to define CE clusters. For this report, the distributions of energies and combined with knowledge of geometrically related pairs residues in true epitopes were analyzed and adopted as variables for CE prediction. The results of our developed system indicate that it provides an outstanding CE prediction with high specificity and accuracy.

## Methods

### CE-KEG workflow architecture

**Figure 1:**
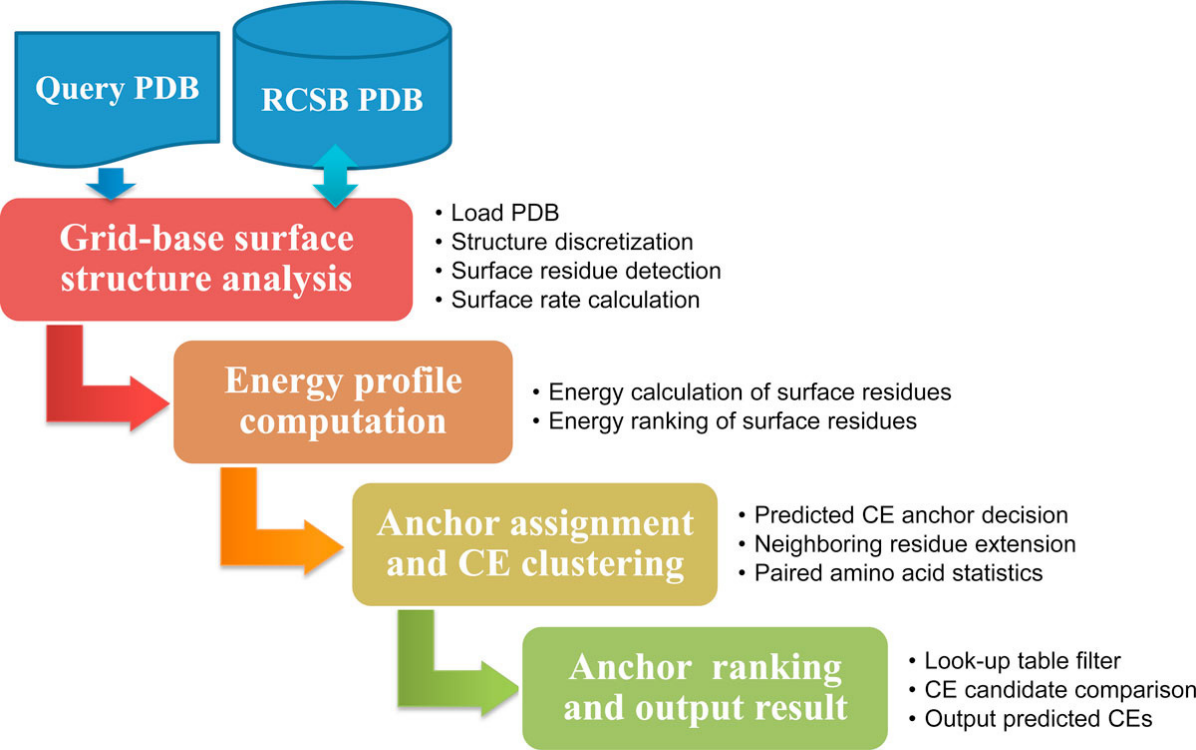
**CE prediction workflow**.

The proposed CE prediction system based on knowledge-based energy function and geometrical neighboring residue contents is abbreviated as "CE-KEG". CE-KEG is performed in four stages: analysis of a grid-based protein surface, an energy-profile computation, anchor assignment, and CE clustering and ranking (Figure 1).

The first module in the "Grid-based surface structure analysis" accepts a PDB file from the Research Collaboratory for Structural Bioinformatics Protein Data Bank [30] and performs protein data sampling (structure discretization) to extract surface information. Subsequently, three-dimensional (3D) mathematical morphology computations (dilation and erosion) are applied to extract the solvent accessible surface of the protein in the "Surface residue detection" submodule [31], and surface rates for atoms are calculated by evaluating the exposure ratio contacted by solvent molecules. Then, the surface rates of the side chain atoms of each residue are summed, expressed as the residue surface rate, and exported to a look-up table. The next module is "Energy profile computation" that uses calculations performed at the ProSA web system to rank the energies of each residue on the targeted antigen surface(s) [28]. Surface residues with greater energies and located at mutually exclusive positions are considered as the initial CE anchors. The third module is "Anchor assignment and CE clustering" which performs CE neighboring residue extensions using the initial CE anchors to retrieve neighboring residues according to energy indices and distances among anchor and extended residues. Additionally, the frequencies of occurrence of pair-wise amino acids are calculated to select suitable potential CE residue clusters. For the final module, "CE ranking and output result" the values of the knowledge-based energy propensities calculated in module 2 and the frequencies of occurrence of the geometrically related residue pairs are weighted and then combined to provide CE predictions.

### Preparation of test datasets

The epitope data derived from the DiscoTope server, the Epitome database, and the Immune Epitope Database (IEDB) were collected to validate the performance of CE-KEG. Using DiscoTope, we obtained a benchmark dataset of 70 antigen-antibody complexes from the SACS database [32]. These complexes had been solved to at least 3-Å resolution, and the antigens contained more than 25 residues. The epitope residues in this dataset were defined and chosen as those within 4 Å of the residues directly bound to the antibody (tied residues). The Epitome dataset contained 134 antigens which were inferred by the distances between the antigens and the complementary-determining of the corresponding antibodies, and these antigens were also successfully analyzed through ProSA's energy function evaluation. Epitome labels residues as interaction sites if an antigen atom is within 6 Å of a complementary-determining antibody region. The IEDB dataset was initially composed of 56 antigen chains acquired at the IEDB website (http://www.immuneepitope.org). This dataset contained only antigens for which the complex-structure annotation "ComplexPdbId" was present in the "iedb_export" zip file. Because 11 of these antigens contained fewer than 35 residues and 2 antigens could not be successfully analyzed by ProSA, we only retained 43 antigen-antibody complexes in the final IEDB dataset. In brief, the total number of testing antigens from previous three resources is 247, and after removing duplicate antigens, a new testing dataset containing 163 non-redundant antigens is used for validation of CE-KEG.

### Surface structure analysis

**Figure 2:**
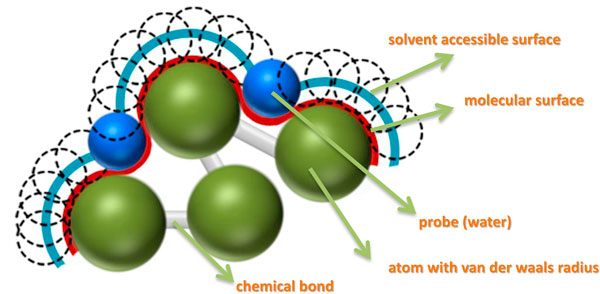
**A cartoon of protein surface representation**.

The interaction between an antigen and an antibody usually depends on their surface resides. The concepts of solvent accessible and molecular surfaces for proteins were first suggested by Lee and Richards [33] (Figure 2). Later, Richards introduced the molecular surface constructs contact and re-entrant surfaces. The contact surface represents the part of the van der Waals surface that directly interacts with solvent. The re-entrant surface is defined by the inward-facing part of a spherical probe that touches more than one protein surface atom [34]. In 1983, Connolly employed the Gauss-Bonnet approach to calculate a molecular surface, which is defined by a small-sized probe that is rolled over a protein's surface [31]. On the basis of the definitions given above, we developed a grid-based algorithm that could efficiently identify surface regions of a protein.

### 3D mathematical morphology operations

Mathematical morphology was initially proposed as a rigorous theoretic framework for shape analysis of binary images. Here, we employed the 3D mathematical morphological dilation and erosion operations for surface region calculations. Based on superior characteristics of morphology in terms of describing shape and structural characteristics, an efficient and effective algorithm was designed to detect precise surface rates for each residue. The query antigen structure was denoted as  X as an object in a 3D grid:

$$X=\left\{\text{v}:f\left(\text{v}\right)=1,\text{v}=\left(x,y,z\right)\in {\text{Z}}^{3}\right\}.$$

where  f is called as the characteristic function of  X. On the other hand, the background $X^{c}$ is defined as:

$$X^{c}=\left\{\text{v}:f\left(\text{v}\right)=0,\text{v}=\left(x,y,z\right)\in {\text{Z}}^{3}\right\}.$$

A 1.5-Å radius sphere is employed as a fundamental structure element *B*. The symmetric of *B *with respect to the origin (0, 0, 0) is denoted as $B^{s}$ and written as

$$B^{s}=\left\{-\text{v}:\text{v}\in B\right\}.$$

The translation of *B *by vector *d *is denoted $B_{d}$ and performed as

$$B_{d}=\left\{\text{v}+\text{d}:\text{v}\in B\right\}.$$

The three elementary morphological operators listed below are then applied for the surface region calculation.

Dilation: $X_{D}=X⊕B_{1}^{S}=\left\{\text{v}\in Z^{3}:B_{1_{\text{v}}}\cap X\neq \emptyset \right\}$

Erosion: $X_{E}=X_{D}⊖B_{2}^{S}=\left\{\text{v}\in {\text{Z}}^{3}:{B_{2}}_{\text{v}}\subset X_{D}\right\}$

**Figure 3:**
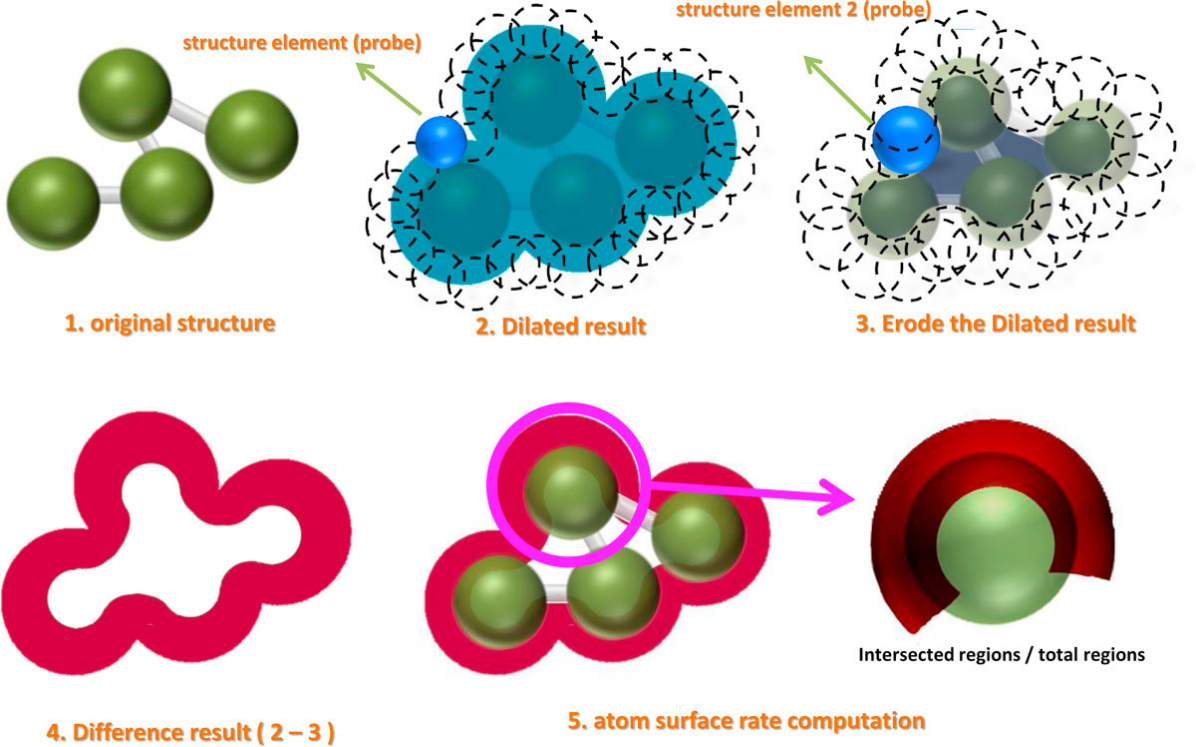
**3D morphology operations used for surface rate calculations**. Shown in the figure are the original, dilated, and eroded structures, the difference between the dilated and eroded structures, and the final atomic surface region.

Difference: $X_{D}-X_{E}$

where the  X is the original structure, $X_{D}$ is a dilated structure by the structuring element $B_{1}$, $X_{E}$ denotes the eroded structure from $X_{D}$ by a larger structuring element $B_{2}$ compared to $B_{1}$, and the surface regions can be achieved by taking difference between $X_{D}$ and $X_{E}$. The surface rate for each atom is obtained by calculating the ratio of the intersected and non-intersected regions with respect to the overlapping areas between the morphological difference operations and the original protein atoms. Figure 3 depicts the step-by-step procedure used to extract the surface regions and to calculate the surface rate for an atom.

### Surface rate computations

The properties of the side chains of the residues in an epitope are important factors controlling protein-protein interactions. Much literature deals with the influence of side chains as factors affecting protein binding. Antigen-antibody binding may cause conformational changes in the proteins, and amino acids that have flexible side chains may, therefore, have an advantage. Experimentally, nonpolar-nonpolar and polar-polar side chain interactions stabilize protein interfaces [35]. Therefore, we considered side chain characteristics in our workflow. With the use of 3D mathematical morphology operations, the rate of each atom, *AR(r)*, can be determined although only the rates of surface side-chain were considered. The surface rate of each residue is denoted *SR*(*r*) and calculated as:

$$SR\left(r\right)=\left\{i\in R:\frac{1}{N}\overset{N}{\underset{i=1}{\sum }}AR\left(r\right)\right\}$$

where *i *represents the *i*^th ^surface atom in the side chain of a residue, *R *is all surface atoms in a residue, and *N *is the total number of surface atoms in residue "*r*".

**Figure 4:**
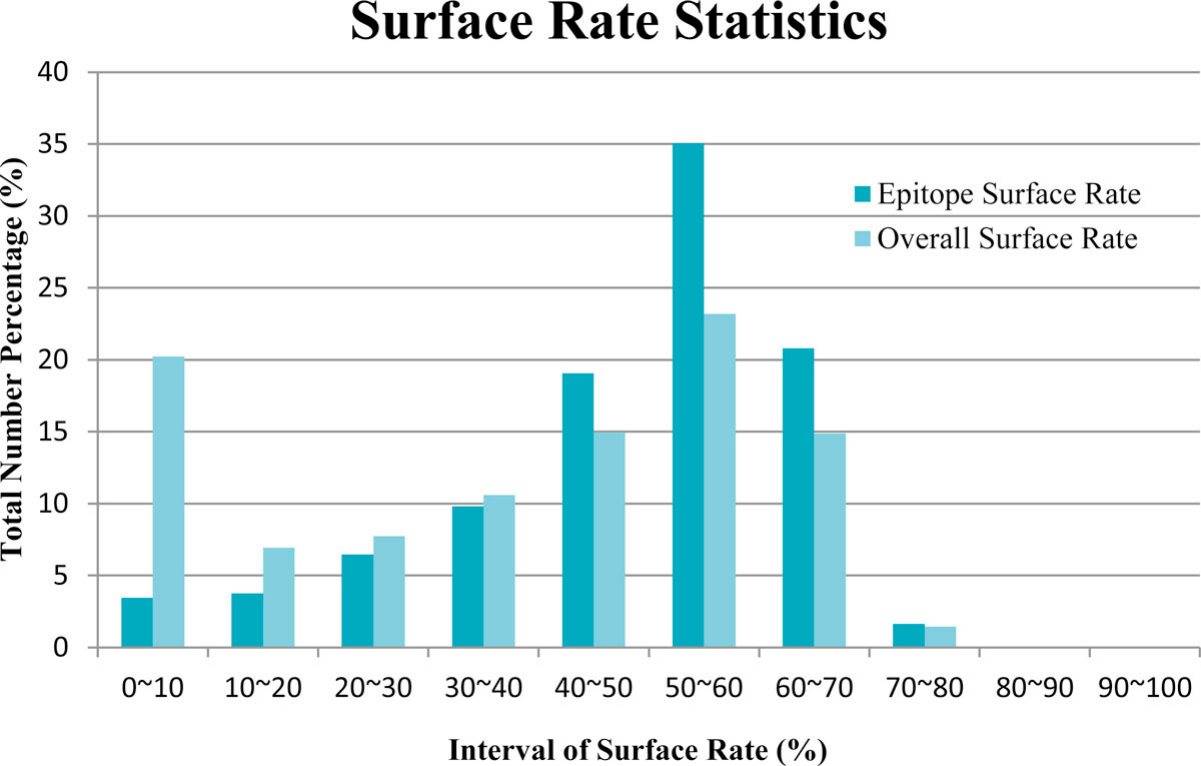
**The distribution of surface rates for residues in known CE epitopes and all surface residues in the antigen dataset**.

Using the equation given directly above, statistics for the surface rates of verified epitope residues and of all surface residues in the non-redundant dataset were acquired, and their distributions are illustrated in Figure 4, which shows that the side chains of residues of known CEs often possessed higher surface rates than do the averaged total areas of the antigens. After calculating the surface rates, they were imported into a file, and a minimum threshold value for the surface rate was set to be used in the predictive workflow.

### Energy profile computation

We used the knowledge-based approach to calculate the energy of each surface residue [28], in conjunction with the distribution of pairwise distances to extract the effective potentials between residues. The potential energy of each residue was calculated using a heavy-atom representation, with the heavy atoms categorized according to the residue in which they were found. The potential calculation represents the ratio between the observed and expected number of contacts for a pair of heavy atoms within a specified distance. The potential value for two atoms reflects the level of attractive interaction between the two residues. Although this knowledge-based potential has usually been used to improve fold recognition, and structure prediction and refinement, we adopted to calculate the energy of each surface residue so as to distinguish among active state conditions. To assess differences in the potentials of CE and non-CE residues, we calculated their surface energy profiles under a variety of parameter settings for 247 known antigens. We found that CE residues possess a higher energy function than do non-epitope residues. When the window size was set to eight residues, the average energy for each verified CE residue cluster in an antigen from the Epitome, DiscoTope, and IEDB datasets was 69.4%, 82.9%, and 51.2% greater than the average energy of non-CE residues in the same antigen, respectively. We also observed that at least one CE residue in each antigen had an energy that was in the top 20% of all surface residues, and most of the largest energies for the CE residues ranked in the top 3%. Therefore, we selected the 20% of the residues with the greatest energies as our initial CE anchors. Additionally, the selected initial seeds were required to possess surface rates within the distribution range of 20% to 50% shown in Figure 4. We also specified that the anchor residues should be separated by at least 12-Å to eliminate possible overlapping CE candidates. With the identities of the initial seeds decided, the relationship between geometrically related neighboring residues within a 10-Å radius sphere of the anchor residue were examined.

### Frequency of occurrence of geometrically related residue pairs

The filtering mechanism used was adopted from a suggestion by Chen that involves the use statistical features for CE verification [29]. However, unlike Chen's proposal that used pairs of sequential residues, CE-KEG incorporated geometrically related neighboring residue pairs. Table 1 shows variables used for the statistical analysis of the residue pairs. Because there are 20 different amino acids, 210 possible unique combinations of pairs are possible, for which we determined the number of times that they were found within CEs and non-CEs. Additionally, the residue pairs found more frequently within spheres of various radii ranging from 2 Å to 6 Å were analyzed respectively, and their corresponding CE indices (CEIs) were also calculated for default settings.

**Table 1:** Variables used in the statistical analysis of geometrically related amino acid pairs (GAAP).

Variables	Description
$N_{GAAP}^{+}$	The number of times a geometrically related residues pair occurs in the known CE epitope dataset.

$N_{GAAP}^{-}$	The number of times a geometrically related amino acid pair occurs in the non-CE epitope dataset.

$f_{GAAP}^{+}$	The frequency (%) that a geometrically related amino acid pair occurs in the known CE epitope dataset.

$f_{GAAP}^{-}$	The frequency (%) that a geometrically related amino acid pair occurs in the non-CE epitope dataset.

$Total_{GAAP}^{+}$	The total number of times that all geometrical amino acid pairs occur in the known CE epitope dataset.

$Total_{GAAP}^{-}$	The total number of times that all geometrical amino acid pairs occur in the non-CE epitope dataset.

$\text{CE}{\text{I}}_{GAAP}$	CEI for a geometrically related amino acid pair.

The CE Index (CEI*_GAAP_*) was obtained by calculating the frequency of occurrence that a pair of geometrically related amino acid in the CE dataset divided by the frequency that the same pair in the non-CE epitope dataset. This value was converted into its log_10 _value and then normalized. For example, the total number of all geometrically related residue pairs in the known CE epitopes is 2843, and the total number of geometrically related pairs in non-CE epitopes is 36,118 when the pairs of residues were within a sphere of radius 2 Å. The two greatest CEIs are for the residue pairs H/Q (0.921) and E/H (0.706) found in from the 247 antigens.

**Figure 5:**
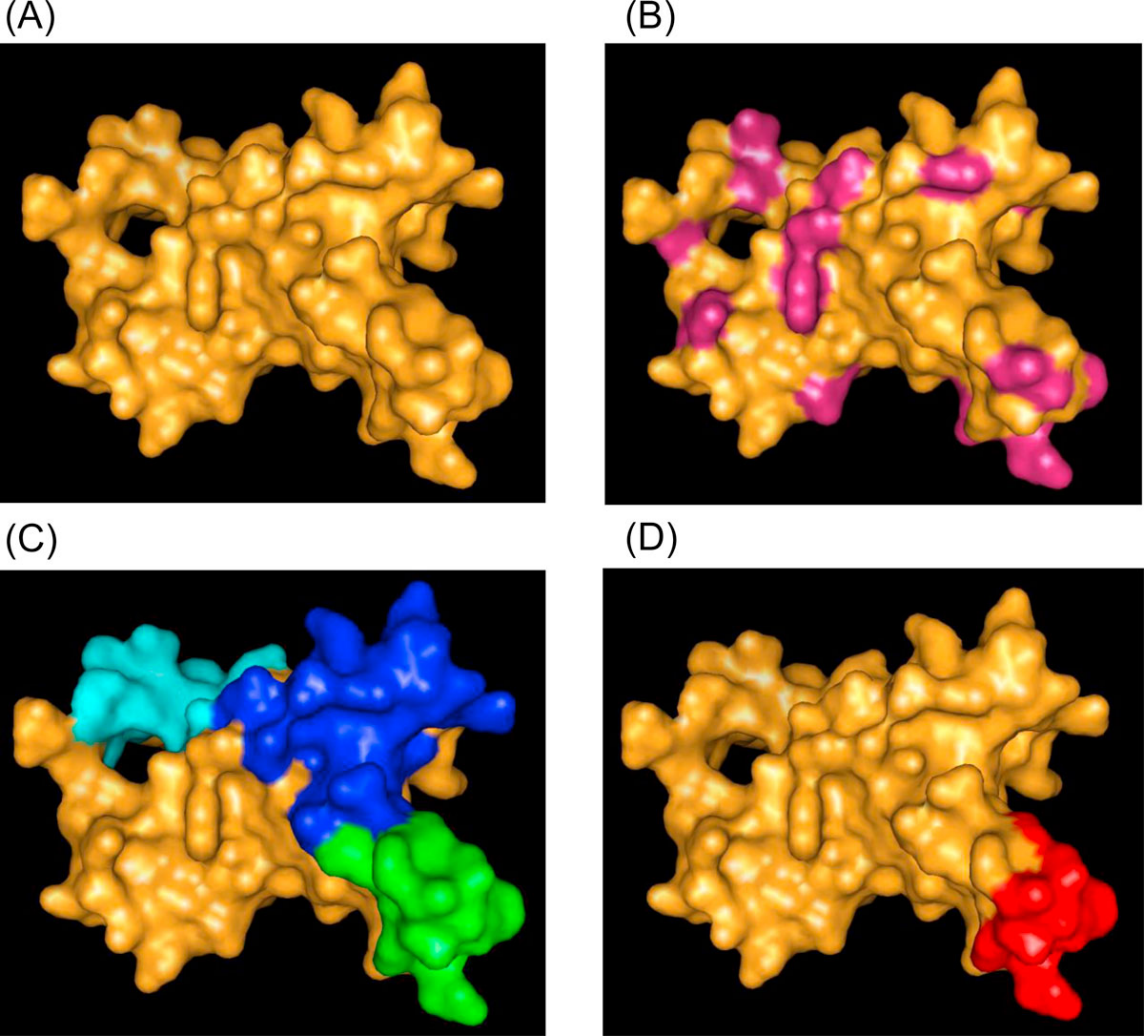
**Example of predicted CE clusters and true CE**. (A) Protein surface of KvAP potassium channel membrane protein (PDB ID: 1ORS:C). (B) Surface seed residues possessing energies within the top 20%. (C) Top three predicted CEs for 1ORS:C. Predicted CEs were obtained by filtering, region growing, and CE cluster ranking procedures. The filtering step removing neighboring residues located within 12 Å according to the energy ranked seed. Region growing formulated the CE cluster from previous filtered seed residues to extend neighboring residues within 10 Å radius. CE clusters were ranking by calculating the combination of weighted CEI and Energy scores. (D) Experimentally determined CE residues.

After determining the CEI for each pair of residues, those for a predicted CE cluster were summed and divided by the number of CE pairs within the cluster to obtain the average CEI for a predicted CE patch. Finally, the average CEI was multiplied by a weighting factor and used in conjunction with a weighted energy function to obtain a final CE combined ranking index. On the basis of the averaged CEI, the prediction workflow provides the three highest ranked predicted CEs as the best candidates. An example of workflow is shown in Figure 5 for the KvAP potassium channel membrane protein (PDB ID: 1ORS:C) [36]. Protein surface delineation, identification of residues with energies above the threshold, predicted CE clusters, and the experimentally determined CE are shown in Figure 5a, b, c, and 5d, respectively.

## Results

In this report, we present a new CE predictor system called CE-KEG that combine an energy function computation for surface residues and the importance of occurred neighboring residue pairs on the antigen surface based on previously known CEs. To verify the performance of CE-KEG, we tested it with datasets of 247 antigen structures and 163 non-redundant protein structures that had been obtained from three benchmark datasets in conjunction with a 10-fold cross-validation assessment. The known CEs had been experimentally determined or computationally inferred prior to our study. For a query protein, we selected the best CE cluster form top three predicted candidate groups and calculated the number of true CE residues correctly predicted by our system to be epitope residues (TP), the number of non-CE residues incorrectly predicted to be epitope residues (FP), the number of non-CE residues correctly predicted not to be epitope residues (TN), and the number of true CE residues incorrectly predicted as non-epitope residues (FN). The following parameters were calculated for each prediction using the TP, FP, TN, and FN values and were used to evaluate the relative weights of the energy function and occurrence frequency used during the predictions:

$$\text{Sensitivity}\left(\text{SE}\right)=\text{TP}÷\left[\text{TP}+\text{FN}\right]$$

$$\text{Specificity}\left(\text{SP}\right)=\text{TN}÷\left[\text{TN}+\text{FP}\right]$$

$$\text{Positive}\;\text{Prediction}\;\text{Value}\;\left(\text{PPV}\right)=\text{TP}÷\left[\text{TP}+\text{FP}\right]$$

$$\text{Accuracy}\left(\text{ACC}\right)=\left[\text{TP}+\text{TN}\right]÷\left[\text{TP}+\text{TN}+\text{FN}+\text{FP}\right]$$

Table 2 shows the predictions when the average energy function of CE residues located within a sphere of 8-Å radius and the frequencies of occurrence for geometrically related residue pairs are combined with different weighting coefficients, whereas Table 3 shows the results when the energies of individual residues are considered. The results show that the performance is better when the average energy is used as compared with the energy of single residues are considered. However, both approaches yield a similar performance for sensitivity, specificity, positive prediction value, and accuracy. For sensitivity, the best average energy weighting coefficient is 10%, which is a consequence of the energy function having been applied prior to the CE-anchor-selection step. Therefore, the energy function of the residues will not have an obvious effect on the prediction results. In this case, the initial parameter settings for new target antigen and the following 10-fold verification will apply with these trained combinations.

**Table 2:** Average performance of the CE-KEG for using average energy function of local neighboring residues.

Weighing Combinations	SE	SP	PPV	ACC
0%EG+100% GAAP	0.478	0.831	0.266	0.796
10%EG + 90% GAAP	0.490	0.831	0.273	0.797
20%EG + 80% GAAP	0.492	0.831	0.275	0.797
30%EG + 70% GAAP	0.497	0.831	0.277	0.798
40%EG + 60% GAAP	0.493	0.832	0.280	0.799
50%EG + 50% GAAP	0.503	0.834	0.284	0.801
60%EG + 40% GAAP	0.504	0.834	0.284	0.801
70%EG + 30% GAAP	0.519	0.839	0.294	0.808
80%EG + 20% GAAP	***0.531***	0.840	0.300	0.811
90%EG + 10% GAAP	0.521	0.839	0.294	0.809
100%EG + 0% GAAP	0.496	0.837	0.279	0.805

The performance used combinations of weighting coefficients for the average energy (EG) and frequency of geometrically related pairs of predicted CE residues (GAAP) within a 8-Å radius sphere. The highest SE is denoted by a bold-italic face.

**Table 3:** Average performance of the CE-KEG for energy function of single residue.

Weighting Combinations	SE	SP	PPV	ACC
0%EG+100% GAAP	0.478	0.831	0.266	0.796
10%EG + 90% GAAP	0.463	0.827	0.260	0.790
20%EG + 80% GAAP	0.473	0.827	0.265	0.791
30%EG + 70% GAAP	0.476	0.828	0.268	0.792
40%EG + 60% GAAP	0.483	0.832	0.275	0.796
50%EG + 50% GAAP	0.466	0.831	0.273	0.795
60%EG + 40% GAAP	0.476	0.833	0.280	0.797
70%EG + 30% GAAP	***0.485***	0.832	0.281	0.797
80%EG + 20% GAAP	0.480	0.830	0.278	0.796
90%EG + 10% GAAP	0.481	0.831	0.275	0.797
100%EG + 0% GAAP	0.463	0.830	0.265	0.795

The performance used combinations of weighting coefficients for the energy (EG) of individual residues and the frequency of occurrence for geometrically related pairs (GAAP). The highest SE is denoted by a bold-italic face.

To evaluate CE-KEG, we adopted a 10-fold cross-validation test. The 247 antigens derived from the DiscoTope, Epitome, and IEDB datasets and the 163 non-redundant antigens were tested as individual datasets. These datasets were randomly partitioned into 10 subsets respectively. Each partitioned subset was retained as the validation proteins for evaluating the prediction model, and the remaining 9 subsets were applied as training data for setting best default parameters. The cross-validation process is repeated for ten times and each of the ten subsets was applied exactly once as the validation subset. The final measurements were then obtained by taking average from individual ten prediction results. For the set of 247 antigens, the CE-KEG achieved an average sensitivity of 52.7%, an average specificity of 83.3%, an average positive prediction value of 29.7%, and an average accuracy of 80.4%. For the set of non-redundant 163 antigens, the average sensitivity was 47.8%; the average specificity was 84.3%; the average positive prediction value was 29.9%; and the average accuracy was 80.7%. For these two datasets, the number of CE clusters assessed was three top predicted ones.

## Discussion and conclusion

With the rapidly increasing number of solved protein structures, CE prediction has become a necessary tool preliminary to wet biomedical and immunological experiments. For the work reported herein, we developed and tested a novel workflow for CE prediction that combines surface rate, a knowledge-based energy function, and the geometrical relationships between surface residue pairs. Because certain existing CE prediction systems do not allow the user to evaluate the values of area under receiver operating characteristic curve (AUC) by altering the parameter settings, an alternatively approximate evaluation of the AUC can be made using the average of the specificity and sensitivity [21]. For example, in comparison with the prediction performance of the DiscoTope system using the DiscoTope benchmark dataset (70 antigens), our workflow provides a better average specificity (83.2% vs. 75%), and a better average sensitivity (62.0% vs. 47.3%). Hence, the AUC value (0.726) returned by CE-KEG is superior to that found for DiscoTope (0.612). To compare CE-KEG with PEPITO (BEPro) system, we used both the Epitome and DiscoTope datasets. The PEPITO system returning averaged AUC values of 0.683 and 0.753, respectively, which are comparable with AUC values of 0.655 and 0.726, respectively returned by CE-KEG. The average number of predicted CEs by employing CE-KEG is approximately six with the most likely predicted CEs ranked at an average position of 2.9. This finding was why we included the top three CEs in our subsequent analysis. Because CE-KEG limits the distance when extending neighboring residues, it predicts CEs that contain a relatively small number of residues. Therefore, CE-KEG performs better than the other tested systems in terms of specificity; however, the sensitivity value is decreased. Future research could focus on the distributions of various physicochemical propensities for epitope and non-epitope surfaces such as the specific geometrical shapes of antigen surfaces, and the unique interactions between antigens and antibodies. Such information may facilitate the appropriate selection of initial CE anchors and provide precise CE candidates for immunological studies.

## Competing interests

The authors declare that they have no competing interests.

## Authors' contributions

YTL and WKW designed the algorithms and performed the experimental data analysis. TWP and HTC conceived the study, participated in its design and coordination, and helped to draft the manuscript. All authors have read and approved the final manuscript.

## Declarations

The funding for publication of this article is provided by the Center of Excellence for Marine Bioenvironment and Biotechnology in National Taiwan Ocean University and National Science Council, Taiwan, R.O.C.

This article has been published as part of *BMC Bioinformatics *Volume 14 Supplement 4, 2013: Special Issue on Computational Vaccinology. The full contents of the supplement are available online at http://www.biomedcentral.com/bmcbioinformatics/supplements/14/S4
